# *Cynanchum wilfordii* Ameliorates Testosterone-Induced Benign Prostatic Hyperplasia by Regulating 5α-Reductase and Androgen Receptor Activities in a Rat Model

**DOI:** 10.3390/nu9101070

**Published:** 2017-09-27

**Authors:** Gyuok Lee, Jawon Shin, Hakjoon Choi, Ara Jo, SangO Pan, Donghyuck Bae, Yongwook Lee, Chulyung Choi

**Affiliations:** 1 Jeonnam Institute of Natural Resources Research, Jangheung-gun, Jeollanamdo 59338, Korea; yellowlgo@naver.com (G.L.); sjo8127@naver.com (J.S.); ohchj12@naver.com (H.C.); joara9153@naver.com (A.J.); jacovan77@gmail.com (S.P.); bdhyuch@naver.com (D.B.); 2 Herbal Hormone Research Institute, Naturalendo Tech Co., Ltd., Gyeonggido, Seongnam 13486, Korea; ywlee@naturalendo.co.kr

**Keywords:** *Cynanchum wilfordii*, benign prostatic hyperplasia, 5α-reductase, androgen receptor, finasteride

## Abstract

Benign prostatic hyperplasia (BPH) is characterized by uncontrolled proliferation of the prostate gland. *Cynanchum wilfordii* has been reported to improve sexual behavior in male rats. In this study, we investigated the protective effect of an aqueous extract of *C. wilfordii* (CWW) against BPH development in a testosterone-induced BPH rat model. The rats were divided into the following six groups: sham/vehicle; BPH/vehicle; BPH/finasteride; and three CWW doses (50, 100, and 200 mg/kg). After a 4-week treatment with CWW, the rats were euthanized at scheduled times, and their prostates were weighed, followed by a histopathological examination. Prostate growth inhibition rates in rats administered CWW 50, 100, and 200 mg/kg were 54.5%, 51.8%, and 50.1%, respectively. The BPH/CWW group showed decreased serum testosterone and dihydrotestosterone (DHT) levels compared to the BPH/vehicle group. Furthermore, the BPH/CWW group showed reduced prostate testosterone and DHT levels compared to the BPH/vehicle group. Mechanistically, the reverse transcription-polymerase chain reaction revealed downregulated mRNA expression levels of the androgen receptor, 5α-reductase, and B-cell lymphoma-2 (Bcl-2) in the BPH/CWW200 group compared with those in the testosterone-induced groups. In conclusion, these findings show the effectiveness of CWW in slowing the progression of testosterone-induced BPH in rats.

## 1. Introduction

Lower urinary tract symptoms (LUTS)/benign prostatic hyperplasia (BPH) commonly occur in older men. The prevalence of BPH is approximately 40% in men in their 50s and reaches 90% in men in their 80s or above [1]. The incidence of LUTS is approximately 25% in men ≥50 years old [2]. BPH is characterized by hyperplasia of the mesenchymal stromal and glandular epithelial cells in the prostate, which results in increased prostate weight and size [3,4]. However, the mechanism underlying the pathogenesis of BPH has not been elucidated.

Most researchers consider androgens as a risk factor in the development and growth of the prostate gland [5,6,7]. Dihydrotestosterone (DHT), which is produced from circulating testosterone by 5α-reductase in the prostate, is an acute mediator of benign prostatic enlargement [8]. Androgens such as prostatic testosterone and DHT exert their physiological effects by binding to the androgen receptor (AR) and regulating AR transcriptional activity.

In addition to prostatectomy, the current treatments for LUTS/BPH are targeted at reducing symptoms and suppressing the progression of the disease [9,10]. In clinical trials, the 5α-reductase inhibitors (5ARIs) used to treat symptomatic BPH significantly suppressed prostate growth and reduced the levels of DHT in the prostate tissue [11]. Finasteride is a commercially available 5α-reductase inhibitor drug used in the treatment of BPH [12]. However, its use is limited because of associated side effects such as dizziness, loss of libido, erectile dysfunction, and upper respiratory tract infection [13,14]. Therefore, plant-derived agents that are effective against BPH development and with fewer side effects should be developed.

Low testosterone levels, such as that observed in andropause, are known to induce rapid cell death in the prostate by activating apoptosis, which reduces the prostate size [15,16,17]. Apoptosis-induced cell death is known to be mediated by a genetically regulated process that requires the expression and subsequent action of discrete gene products and coregulatory molecules such as B-cell lymphoma-2 (Bcl-2), an inner mitochondrial membrane protein, and caspase-3 proteins [18].

*Cynanchum wilfordii* is used in traditional herbal medicine in Korea for the prevention and treatment of various diseases such as rheumatic arthritis, geriatric diseases, atherosclerotic vascular diseases, and ischemia-induced diseases [19]. Research is ongoing on the use of *C. wilfordii* to ameliorate menopausal symptoms [20]. In addition, our previous study reported that *C. wilfordii* improved sexual behavior in male rats [21]. Although BPH, a major cause of male sexual dysfunction, has been actively studied, no investigation has reported the efficacy of *C. wilfordii* against BPH. Therefore, we aimed to find a natural product for the treatment of BPH by investigating the effects of *C. wilfordii* in a testosterone-induced rat model of BPH and elucidate the mechanisms underlying its activity.

## 2. Materials and Methods

### 2.1. Sample Procurement

*C. wilfordii* roots were procured from Naturalendo Tech (Seongnam, Korea), identified by one of the authors (Yongwook Lee, Ph.D.), and a voucher specimen was deposited in the Herbarium of Jeonnam Institute of Natural Resources Research (Voucher specimen No. CW1504). *C. wilfordii* roots were extracted with distilled water at 100 °C for 4 h, the extract (CWW) was filtered, concentrated under vacuum (Buchi, Flawil, Switzerland), lyophilized (Ilshinbiobase, Dongducheon-si, Korea), and subsequently used for the experiments.

### 2.2. Experimental Animals and Maintenance Conditions

Male Sprague-Dawley rats (3-month-old, weighing 370–390 g) were purchased from Samtako Bio Co., (Osan, Korea). After a 1-week acclimatization, the animals were housed in pairs in plastic cages under a 12-h light/dark cycle in rooms maintained at 18–23 °C and 4–60% humidity. Water and standard laboratory diet were provided ad libitum. This study was approved by the Institutional Animal Care and Use Committee of the Jeollanamdo Institute for Natural Resources Research (confirmation number: JINR1601).

### 2.3. Orchiectomy Procedure

The rats were divided into the following six groups, which were treated as indicated: sham/vehicle, BPH/vehicle, BPH/finasteride, and three CWW doses (50, 100, and 200 mg/kg). To prevent the influence of endogenous testosterone, rats in all the groups except the sham group underwent bilateral orchiectomy 3 days prior to testosterone treatment. For the orchiectomy, the animals were anesthetized by intraperitoneal injections of Zoletil 50 (Virbac, Nice, France). The sham group rats underwent the same procedure as those in the other groups except that their testicles were not removed. Rats in the BPH-induced groups were castrated. Briefly, the testes were exposed by transverse resecting of both scrota in the supine position. Then, the spermatic cord and blood vessels were ligated with 3-0 sutures and resected.

### 2.4. Induction of BPH 

The animals were randomly assigned to either the sham or BPH-induced groups. Prostatic hyperplasia was induced in the BPH-induced group by daily subcutaneous (s.c.) injections of testosterone (20 mg/kg, Tokyo Chemical Industry, Tokyo, Japan) for 4 weeks, as reported previously [22]. The testosterone was dissolved in sterile corn oil for s.c. injection. At the end of the experiment, the sham and BPH-induced groups were anesthetized under light ether (Merck, Dormstad, Germany) anesthesia and killed by cervical dislocation. The prostate was removed from all animals. Each prostate was weighed and prepared for further analysis.

### 2.5. Sample Administration Groups

Following the induction of prostatic hyperplasia, orchiectomized rats were randomly divided into six groups (*n* = 9) that received respective treatments daily for 4 weeks. CWW and finasteride were dissolved in phosphate-buffered saline (PBS) for oral administration. The dosage of CWW was adjusted weekly based on the body weight change. All solutions were freshly prepared before each experimental series. All preparations were administered orally in a 0.2-mL volume using a gavage needle (JD-S-124, Jeungdo bio, nowon, Korea) once a day for 4 weeks. The animals were grouped, and the sham/vehicle group was administered PBS orally and s.c. injections of corn oil. All the other groups were administered s.c. testosterone (20 mg/kg) injections in addition to the following treatments: BPH/vehicle, PBS orally; BPH/finasteride, finasteride (5 mg/kg, Tokyo Chemical Industry, Tokyo, Japan) orally; and the BPH/CWW groups, CWW (50, 100, and 200 mg/kg) by oral gavage. The positive control drug finasteride is a well-known 5α-reductase inhibitor used for BPH treatment. The effective dose of finasteride was selected based on the results of a previous study [23]. Body weight was measured weekly during the experiment. The experiment was carried out for 4 weeks. Blood samples were drawn from the caudal vena cava, the serum was separated by centrifugation (I-15PK, Sartorius, Göttingen, Germany), and then stored at −80 °C. The whole prostates were immediately removed and weighed, and then 4–5-μm thick sections of the ventral prostate lobe were cut, and then fixed in 10% neutral-buffered formalin, followed by embedment in paraffin for histological analysis. The remainder of each prostate was stored at −70 °C and used to evaluate the hormone (testosterone and DHT) levels.

### 2.6. Prostate Weight

Prostate tissues were harvested and weighed immediately. Then, the percentage growth inhibition was calculated as follows:

$$\mathrm{Growth}\;\mathrm{inhibition}\;\left(\%\right)=100-\left(\frac{\mathrm{Treated}\;\mathrm{group}-\mathrm{sham}\;\mathrm{group}}{\mathrm{BPH}\;\mathrm{group}-\mathrm{sham}\;\mathrm{group}}\times 100\right)$$

### 2.7. Determination of Testosterone and DHT Levels in Serum and Prostate

The prostate tissue samples were homogenized (1/10 *w*/*v*) using a homogenizer in a tissue lysis/extraction reagent containing a protease inhibitor cocktail (Sigma-Aldrich; Merck Millipore, Darmstadt, Germany). The homogenates were centrifuged at 12,000× *g* for 25 min at 4 °C, and the protein concentration in the supernatant fractions was determined using the Bradford reagent (Bio-Rad Laboratories, Inc., Hercules, CA, USA). Testosterone and DHT levels in the serum and prostate tissue were measured using an enzyme-linked immunosorbent assay (ELISA) kit (Cusabio Biotech, Wuhan, China). The values were expressed as milligram protein and milliliters for the prostate and serum, respectively.

### 2.8. Determination of Prostate-Specific Antigen (PSA) Levels in Serum 

Prostate-specific antigen (PSA) levels in the serum were measured using an ELISA kit (Cusabio Biotech, Wuhan, China). The PSA ELISA is a solid-phase, noncompetitive immunoassay based on the direct sandwich technique. Calibrators, controls, and samples were incubated with the biotinylated anti-PSA monoclonal antibody and horseradish peroxidase (HRP)-labelled anti-PSA monoclonal antibody in streptavidin-coated microtiter strips. After washing, the buffered substrate (3,3′,5,5′-tetramethylbenzidine (TMB)-HRP), which contains hydrogen peroxide and the chromogen reagent (TMB) was added to each well, and the enzyme reaction was allowed to proceed. The color intensity was determined using a microtiter plate spectrophotometer (Molecular devices, Sunnyvale, CA, USA) at 620 nm. Calibration curves were constructed for each assay by plotting the absorbance versus the concentration of each calibrator. The PSA concentration of the samples was then read from the calibration curve and expressed as milliliters in the serum.

### 2.9. Reverse Transcription-Polymerase Chain Reaction (RT-PCR) Analysis of AR, 5α-Reductase, and Bcl-2

Total RNA was isolated using an RNeasy^®^ Mini kit (Qiagen, Hilden, Germany), according to the manufacturer’s protocol. Total RNA (2 μg) from each sample was subjected to reverse transcription (RT) using a QuantiTect^®^ RT kit (Qiagen, Hilden, Germany). The polymerase chain reaction (PCR) assays were conducted using the EmeraldAmp^®^ PCR Master Mix kit (Takara Bio, Inc., Singa, Japan). The following primers were used: AR, 5′-CAAAGGGTTGGAAGGTGAGA-3′ and 5′-GAGCGAGCGGAAAGTTGTAG-3′; 5α-reductase, 5′-GGCATGCAGGGTCATGCCTGCTTAGCC-3′ and 5′-ACCTGCACATGAAACAAACATGCAG-3′; Bcl-2, 5′-AGGATTGTGGCCTTCTTTGAGT-3′ and 5′-GCCAGCACCATGAAGATCAA-3′; and β-actin, 5′-CAGCTCCTCCGTCGCCGGTCCACAC-3′ and 5′-CTGACCCATACCCACCATCACACCC-3′. The reaction (Bio-rad, CA, USA) mixtures were incubated at 95 °C for 120 s, followed by 40 cycles at 95 °C for 30 s and 60 °C for 30 s. The RT-PCR products were analyzed using gel electrophoresis with β-actin as an internal control.

### 2.10. Histological Examination

The prostate tissues were fixed in 4% buffered-paraformaldehyde for 24 h, washed with 70% ethanol, dehydrated with a graded alcohol series, embedded in paraffin, sectioned at 4-μm thickness, and then stained with hematoxylin and eosin (H&E, Sigma-Aldrich, St. Louis, MO, USA). The images were captured using a microscope (Foculus, Leica, Germany).

### 2.11. Measurement of Serum Alanine Aminotransferase (ALT) and Aspartate Aminotransferase (AST) Levels

At the end the experimental period, blood samples were collected, coagulated by maintenance at room temperature for 30 min, and then the serum was separated by centrifugation (3000× *g*, 4 °C, 20 min). The alanine aminotransferase (ALT) and aspartate aminotransferase (AST) levels were determined to assess the liver function using the FUJI DRI-CHEM SLIDE kit and the FUJI DRI-CHEM 4000 analyzer (Fujifilm, Tokyo, Japan).

### 2.12. Standardization of CWW

The CWW was standardized based on the 4-hydroxyacetophenone and 2,4-hydroxyacetophenone content of the extract, which was determined using a high-performance liquid chromatography (HPLC) system (Shimadzu UFLC(XR)-20A, Kyoto, Japan) with a Triart C18 plus (250 mm × 4.6 mm, 5-μm). The mobile phase, which consisted of solvent A (methanol) and B (0.1% formic acid), was run at a flow rate of 1 mL/min. The solvent system flow gradient mode was as follows: 0–5 min, 20% A; 5–12 min, 20–30% A; 12–20 min, 30–40% A; 20–25 min, 40–80% A; 25–35 min, 80–100% A; 35–46 min, 100% A; 46–50 min, 100–20% A; 50–60 min, 20% A; and finally, the column was washed and reconditioned. The sample injection volume was 20 μL while the measurements were performed at a wavelength of 284 nm and 35 °C using optimum temperatures for the HPLC separation.

### 2.13. Statistical Analysis

The results are expressed as the mean ± standard error of the mean (S.E.M.) Comparison between groups was performed using a one-way analysis of variance (ANOVA). Differences between individual treatment groups were compared using Dunn’s test. Statistical significance was accepted at *p <* 0.05 and the statistical analyses were performed using the GraphPad Prism software version 5.0 (GraphPad Software, Inc., La Jolla, CA, USA) for Windows.

## 3. Results

### 3.1. Effects of CWW on Body and Prostate Weights

The rats were weighed every 7 days. In the BPH/vehicle group, the body weight gain decreased compared with that in the sham/vehicle group. However, the body weight gain in the BPH/CWW and BPH/finasteride groups showed no significant difference with that in the BPH/vehicle groups. The prostate weight is an important indicator of BPH. The BPH/vehicle group showed a significant increase in prostate weight (1.39 ± 0.07 g) compared to that of the sham/vehicle group (Table 1). The BPH/finasteride group showed a significant reduction in prostate weight (1.06 ± 0.06 g, *p <* 0.01) compared to the BPH/vehicle group. The BPH/CWW 50, 100, and 200 groups showed significant reductions in prostate weight (1.08 ± 0.07, 1.10 ± 0.05, and 1.11 ± 0.08 g, respectively, *p <* 0.05) compared to the BPH/vehicle group. Regarding growth inhibition, the BPH/finasteride group showed the most effective reduction in growth (58.1%).

### 3.2. Effects of CWW on Serum Testosterone and DHT Levels

**Figure 1:**
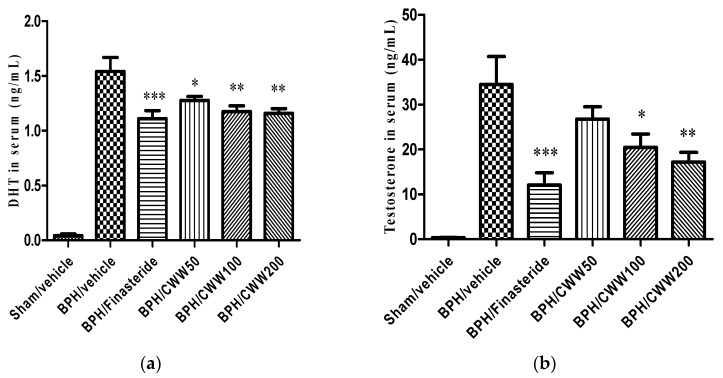
Effects of aqueous *Cynanchum wilfordii* extract (CWW) on serum (**a**) dihydrotestosterone (DHT) and (**b**) testosterone levels. *** *p <* 0.005, ** *p <* 0.01, and * *p <* 0.05 vs. the BPH/vehicle group. BPH: benign prostatic hyperplasia; Sham/vehicle: sham-operated and phosphate-buffered saline (PBS), oral (p.o.); BPH/vehicle: testosterone, subcutaneous (s.c.) and PBS, p.o.; BPH/finasteride: testosterone, s.c. and finasteride 5 mg/kg, p.o.; BPH/CWW 50: testosterone, s.c. and CWW 50 mg/kg, p.o.; BPH/CWW 100: testosterone, s.c. and CWW 100 mg/kg, p.o.; BPH/CWW 200: testosterone, s.c. and CWW 200 mg/kg, p.o.

The main prostatic androgen is DHT. The BPH/vehicle group showed a significant increase in serum testosterone levels compared with the sham/vehicle group (34.46 ± 6.26 and 0.37 ± 0.04 ng/mL, respectively). However, the BPH/finasteride (12.08 ± 2.73 ng/mL) and BPH/CWW 50, 100, and 200 (26.79 ± 2.73, 20.48 ± 2.98, and 17.2 ± 2.15 ng/mL, respectively) showed significantly decreased serum testosterone levels compared to that of the BPH/vehicle group (*p <* 0.05, Figure 1b). Serum DHT levels in the BPH/vehicle group (1.54 ± 0.13 ng/mL) increased significantly compared to those in the sham/vehicle group (0.04 ± 0.02 ng/mL). However, serum DHT levels in the BPH/finasteride (1.11 ± 0.07 ng/mL) and CWW 50-, 100-, and 200-treated groups (1.28 ± 0.04, 1.18 ± 0.05, and 1.16 ± 0.04 ng/mL, respectively) decreased significantly compared with those in the BPH/vehicle group (*p <* 0.05, Figure 1a).

### 3.3. Effects of CWW on Prostate Testosterone and DHT Levels

**Figure 2:**
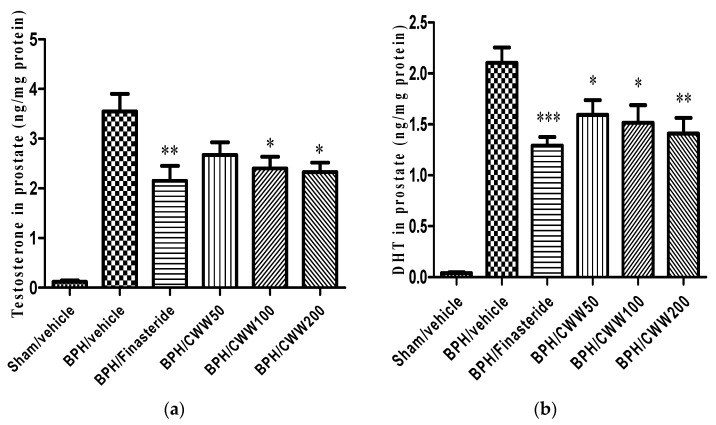
Effects of aqueous *Cynanchum wilfordii* extract (CWW) on prostate (**a**) dihydrotestosterone (DHT) and (**b**) testosterone levels. *** *p <* 0.005, ** *p <* 0.01, and * *p <* 0.05 vs. BPH/vehicle group. BPH: benign prostatic hyperplasia; Sham/vehicle: sham-operated and phosphate-buffered saline (PBS), oral (p.o.); BPH/vehicle: testosterone, subcutaneous (s.c.) and PBS, p.o.; BPH/finasteride: testosterone, s.c. and finasteride 5 mg/kg, p.o.; BPH/CWW 50: testosterone, s.c. and CWW 50 mg/kg, p.o.; BPH/CWW 100: testosterone, s.c. and CWW 100 mg/kg, p.o.; BPH/CWW 200: testosterone, s.c. and CWW 200 mg/kg, p.o.

The prostatic testosterone and DHT levels are shown in Figure 2. The BPH/vehicle group demonstrated increased levels of testosterone and DHT (3.55 ± 0.35 and 2.10 ± 0.15 ng/mL, respectively) compared to the sham/vehicle group while the BPH/finasteride group demonstrated markedly decreased testosterone and DHT (2.15 ± 0.30 and 1.29 ± 0.09 ng/mL, respectively) levels compared to the BPH/vehicle group. Prostatic testosterone levels in the BPH/CWW 100 and BPH/CWW 200 groups decreased significantly compared with those in the BPH/vehicle group (*p <* 0.05). Prostatic DHT levels in the BPH/CWW 50 group did not decrease significantly compared with those in the BPH/vehicle group. Prostatic DHT levels in the BPH/CWW 100 and BPH/CWW 200 groups significantly and dose-dependently decreased compared to the levels in the BPH/vehicle group.

### 3.4. Effects of CWW on Serum PSA Levels

**Figure 3:**
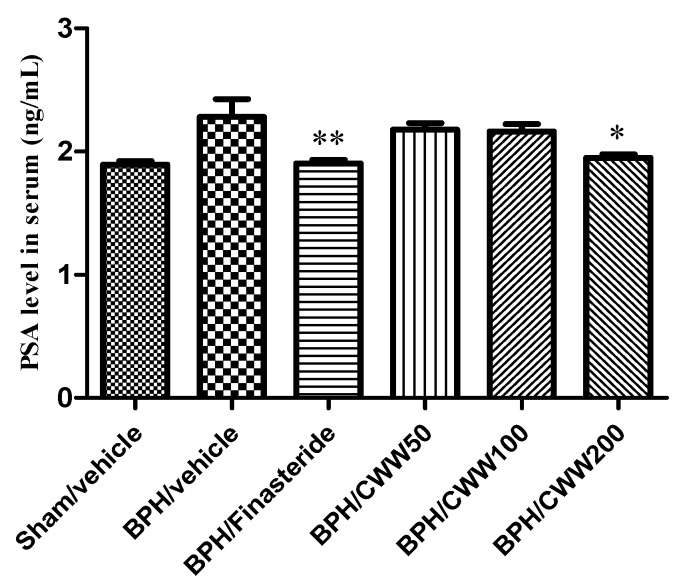
Effects of aqueous *Cynanchum wilfordii* extract (CWW) on serum prostate-specific antigen (PSA) levels. ** *p <* 0.01 and * *p <* 0.05 vs. BPH/vehicle group. BPH: benign prostatic hyperplasia; Sham/vehicle: sham-operated and phosphate-buffered saline (PBS), oral (p.o.); BPH/vehicle: testosterone, subcutaneous (s.c.) and PBS, p.o.; BPH/finasteride: testosterone, s.c. and finasteride 5 mg/kg, p.o.; BPH/CWW 50: testosterone, s.c. and CWW 50 mg/kg, p.o.; BPH/CWW 100: testosterone, s.c. and CWW 100 mg/kg, p.o.; BPH/CWW 200: testosterone, s.c. and CWW 200 mg/kg, p.o.

The BPH/vehicle group demonstrated a markedly increased serum PSA level (2.28 ± 0.14 ng/mL) compared to that of the sham/vehicle group (1.89 ± 0.03 ng/mL, Figure 3). However, the BPH/finasteride group (1.91 ± 0.03 ng/mL) exhibited a significant decrease in serum PSA levels compared to the BPH/vehicle group (*p <* 0.01). The BPH/CWW 200 group (1.95 ± 0.30 ng/mL) demonstrated a significant reduction in PSA levels compared with the BPH/vehicle group (*p <* 0.05).

### 3.5. Effects of CWW on Prostate Tissue Histology

**Figure 4:**
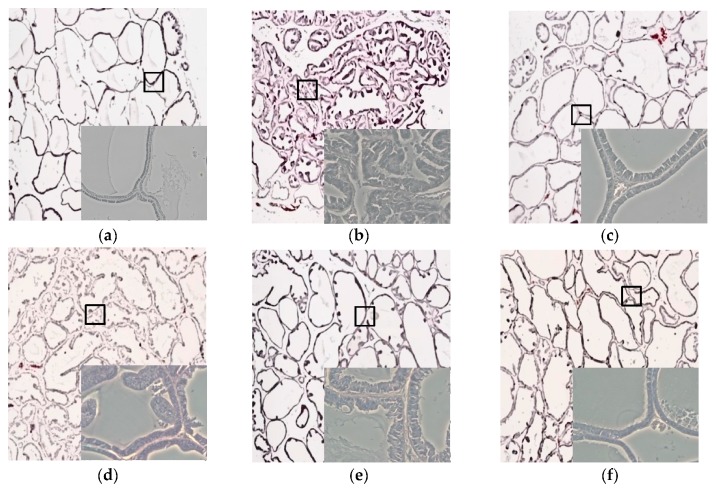
Effects of aqueous *Cynanchum wilfordii* extract (CWW) on prostate hyperplasia. Histological examination of prostate tissues was performed 24 h after the final testosterone injection. Prostate tissues were fixed, sectioned at 4-μm thickness, and stained with hematoxylin and eosin (H&E; magnification, ×10; small box, ×20). BPH: benign prostatic hyperplasia; (**a**) Sham/vehicle: sham-operated and phosphate-buffered saline (PBS), oral (p.o.); (**b**) BPH/vehicle: testosterone, subcutaneous (s.c.) and PBS, p.o.; (**c**) BPH/finasteride: testosterone: s.c. and finasteride 5 mg/kg, p.o.; (**d**) BPH/CWW 50: testosterone, s.c. and CWW 50 mg/kg, p.o.; (**e**) BPH/CWW 100: testosterone, s.c. and CWW 100 mg/kg, p.o.; (**f**) BPH/CWW 200: testosterone, s.c. and CWW 200 mg/kg, p.o.

The tubular glands of the sham/vehicle group were tightly formed, lined with cuboidal epithelium, and supported by a connective tissue stroma (Figure 4a). In contrast, the BPH/vehicle group demonstrated typical features of glandular hypertrophy including increased numbers of acini, nuclear stratification, papillary fronds protruding into the glandular cavities, thickening of the prostatic epithelial layer, and decreased luminal volume (Figure 4b). It was difficult to find a histological difference between the BPH/vehicle and BPH/CWW 50 groups (Figure 4d). However, the BPH/CWW 200 group showed noticeably glandular hyperplasia compared to the BPH/vehicle group. In addition, the glandular luminal area increased, and the prostatic epithelial height and polyp formations decreased significantly (Figure 4f). The BPH/finasteride group also showed reductions in the epithelial thickness (Figure 4c).

### 3.6. Assessment of AR, 5α-Reductase, and Bcl-2 mRNA Expression

**Figure 5:**
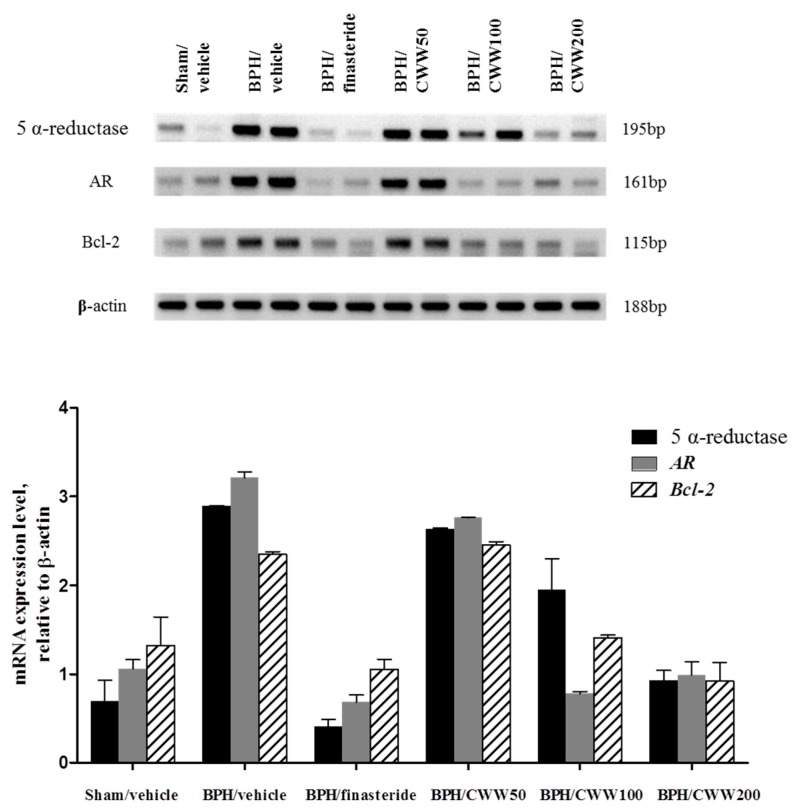
Effects of aqueous *Cynanchum wilfordii* extract (CWW) on mRNA expression of 5α-reductase, AR (Androgen receptor), and B-cell lymphoma-2 (Bcl-2). Data are means ± standard error of the mean (S.E.M., *n* = 2). BPH: benign prostatic hyperplasia; Sham/vehicle: sham-operated and phosphate-buffered saline (PBS), oral (p.o.); BPH/vehicle: testosterone, subcutaneous (s.c.) and PBS, p.o.; BPH/finasteride: testosterone, s.c. and finasteride 5 mg/kg, p.o.; BPH/CWW 50: testosterone, s.c. and CWW 50 mg/kg, p.o.; BPH/CWW 100: testosterone, s.c. and CWW 100 mg/kg, p.o.; BPH/CWW 200: testosterone, s.c. and CWW 200 mg/kg, p.o.

The effects of CWW on AR and 5α-reductase were studied in prostate tissues. Testosterone administration markedly increased the mRNA expression of both AR and 5α-reductase (Figure 5a,b) compared with the sham/vehicle group. Coadministration of BPH/CWW 50 did not reduce the testosterone-mediated increase in mRNA expression (Figure 5a,b). However, the mRNA expression of AR and 5α-reductase decreased markedly in the BPH/finasteride as well as the BPH/CWW 100 and 200 groups. The anti-apoptotic factor Bcl-2 was analyzed using RT-PCR. The BPH/vehicle group showed a marked upregulation of Bcl-2 mRNA expression compared with that of the sham/vehicle group (Figure 5c). However, the testosterone-mediated upregulation of Bcl-2 mRNA expression decreased significantly in the BPH/CWW groups.

### 3.7. Toxicity of CWW in Rat BPH Model

**Figure 6:**
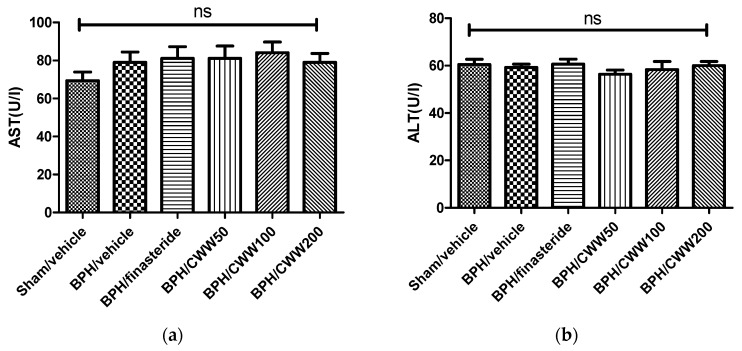
Levels of serum (**a**) aspartate aminotransferase (AST) and (**b**) alanine aminotransferase (ALT). BPH: benign prostatic hyperplasia; Sham/vehicle: sham-operated and phosphate-buffered saline (PBS), oral (p.o.); BPH/vehicle: testosterone, subcutaneous (s.c.) and PBS, p.o.; BPH/finasteride: testosterone, s.c. and finasteride 5 mg/kg, p.o.; BPH/CWW 50: testosterone, s.c. and CWW 50 mg/kg, p.o.; BPH/CWW 100: testosterone, s.c. and CWW 100 mg/kg, p.o.; BPH/CWW 200: testosterone, s.c. and CWW 200 mg/kg, p.o.

As shown in Figure 6, CWW did not promote the activities of the serum enzyme markers (ALT and AST) of liver toxicity, indicating normal liver function.

### 3.8. Standardization of CWW

**Figure 7:**
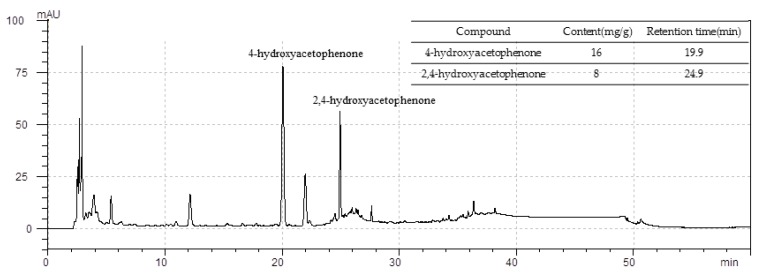
Representative high-performance liquid chromatography (HPLC) chromatograms of aqueous *Cynanchum wilfordii* extract (CWW). Mobile phase consisted of solvents A (methanol) and B (0.1% formic acid) run at a flow rate of 1.0 mL/min. Elution conditions were as follows: 0–5 min, 20% A; 5–12 min, 20–30% A; 12–20 min, 30–40% A; 20–25 min, 40–80% A; 25–35 min, 80–100% A; 35–46 min, 100% A; 46–50 min, 100–20% A; 50–60 min, 20%; and finally, the column was washed and reconditioned. Sample injection volume was 20 μL. Optimum HPLC separation was achieved at 35 °C and monitored at 284 nm. AU: arbitrary unit.

As shown in Figure 7, CWW was standardized based on the 4-hydroxyacetophenone and 2,4-hydroxyacetophenone content, which was determined using HPLC. The standardized CWW relative to the main ingredients 4-hydroxyacetophenone and 2,4-hydroxyacetophenone (both *R*^2^ = 0.9991) indicated good linearity while their retention times were typically detected near 19.9, and 24.9 min, respectively. The mean 4-hydroxyacetophenone and 2,4-hydroxyacetophenone content in the CWW was approximately 16 and 8 mg/g of extract, respectively.

## 4. Discussion

BPH is a multifactorial disease characterized by non-cancerous enlargement and an imbalanced proliferation of smooth muscle and epithelial cells of the prostatic tissue. Clinically, BPH results in LUTS, an age-related detrusor dysfunction with a significant unsatisfactory impact on the quality of life of the patient [24]. BPH progression is dependent on several factors such as stromal-epithelial interactions, adrenergic stimulation, and apoptotic processes, which drive the proliferation [25,26]. The presence of testicular androgens is a well-known risk factor for BPH because androgens, including testosterone and DHT, play a crucial role in regulating cell proliferation and death in the prostate. In the present study, we aimed to investigate the effects of CWW on BPH in a testosterone-induced BPH rat model. We found that the extract was effective in slowing the progression of BPH induced by testosterone.

Testosterone and DHT play a key role in the development of male reproductive organs, and these hormones are commonly associated with BPH [27]. DHT is a more potent androgen than testosterone because of its higher affinity for AR; DHT has three times greater affinity for ARs than testosterone does and 15–30 times greater affinity for the receptor than adrenal androgen [28]. The hormone receptor binds to specific DNA-binding sites in the nucleus to increase the transcription of androgen-dependent genes, and ultimately stimulate protein synthesis [29]. In particular, the expression of DHT, which is the most potent form of prostate testosterone, is correlated with the development of BPH [30]. Overproduction of DHT, which occurs with aging, leads to the development and exacerbation of BPH [31].

Prostatic enlargement is an important marker of BPH [32]. In the present study, we assessed the effects of CWW on prostate weight in a testosterone-induced BPH rat model. The BPH/vehicle group showed increased prostate weight compared with the sham group, and experimentally induced prostatic hyperplasia was observed during the histopathological analysis of the prostate tissues. The BPH/vehicle group showed pathological alterations with stromal proliferation and glandular hyperplasia in the prostate, whereas the BPH/CWW groups demonstrated significant inhibition of the increase in prostate weight. In the BPH/vehicle group, columnar epithelial cells were arranged in multiple layers, and the proliferation of epithelial cells and the number of glands were increased compared with the Sham/vehicle group. Notably, the BPH/CWW 200 group showed similar histological architecture to the BPH/finasteride group, although the improvement in prostate weight and histology were not dose-dependent.

Since DHT is synthesized from testosterone by the steroid 5α-reductase, numerous researchers have aimed to reduce DHT levels by inhibiting 5α-reductase. Finasteride is a 5α-reductase inhibitor and an elective drug used for the treatment of BPH. It reduces testosterone and DHT levels in the serum and prostate, resulting in the reduction of prostate size and, consequently, relieving the LUTS associated with BPH [33]. However, finasteride also produces serious side effects including an increased risk of impotence, erectile dysfunction, decreased libido, and ejaculation disorder. This has led researchers to investigate alternative methods with fewer side effects for treating BPH [13,14].

Previous studies have reported the protective effects of numerous alternative materials against prostatic hyperplasia based on their 5α-reductase inhibitory activity [34]. In the present study, finasteride reduced testosterone and DHT levels in the serum and prostate. These findings are consistent with those of a previous study [35]. In addition, BPH/CWW decreased the levels of testosterone and DHT in the serum and prostate.

On the other hand, the androgen/AR signaling pathway is known to play a key role in the development of BPH and targeting androgen/AR signaling could be a major therapeutic approach for BPH [36]. Binding of DHT to AR leads to the activation of numerous genes that promote prostatic growth [37]. Testosterone-treated rats have been reported to over-express AR [22,38]. AR regulates gene expression by binding androgen, testosterone, or DHT as transcription factors. After AR binds to androgen, it also increases the transcription of androgen genes and protein synthesis. In the present study, testosterone significantly increased serum DHT concentration, upregulated gene expression of *AR*, and induced histopathological alterations in the prostate tissue. In addition, our data revealed that the BPH/vehicle group over-expressed AR. Treatment with CWW 200 decreased the testosterone-induced expression of AR in the prostate.

PSA is produced in the prostate and is normally present in small quantities in the serum [39]. However, PSA levels have been found to increase in BPH and prostate cancer [40]. Therefore, a reduction in PSA level indicates the effectiveness of a test material in the treatment of BPH [41]. Following its binding, androgen activates AR, which in turn causes it to interact with the ARE in the promoter region of target genes including PSA, thereby regulating the transcription of target genes [42]. Collectively, our findings on prostate weights and histopathological examinations indicate that CWW inhibited the occurrence and progression of BPH in the rat model. The results of this study are closely associated with a reduction in testosterone, DHT, and PSA levels. In the present study, the CWW 200 treatment markedly ameliorated testosterone-induced expression of AR and 5α-reductase in the prostate tissue. Moreover, PSA levels increased in BPH-induced rats but reduced markedly in BPH/CWW 200 groups compared with those in the BPH/vehicle group.

Several studies have shown that a critical cell-signaling pathway regulated by c-Fos expression is involved in castration-induced apoptosis [43,44]. This signaling abruptly and transiently alters the synthesis of Fos antigen, p53, Bcl-2-associate X protein (Bax), and Bcl-2 proteins in AR-expressing prostate epithelial cells [45,46,47]. Apoptosis is important for the development and maintenance of tissue homeostasis in multicellular organisms [48,49]. Thus, apoptotic activity has been suggested to be a key cofactor in the development and progression of BPH. Bcl-2 protein has a pivotal role in regulating the mitochondrial apoptosis signaling pathway. It is involved in the regulation of prostate apoptosis and regarded as a potent apoptosis suppressor [50]. A previous study reported that the level of Bcl-2 was highly upregulated in patients with BPH compared to that in patients with normal prostates [51]. In the current study, Bcl-2 expression was higher in the BPH/vehicle rats than it was in the sham rats, and treatment with CWW suppressed the enhancement of Bcl-2 expression compared with that in rats in the BPH/vehicle group. To better understand the molecular mechanism underlying these actions, more research would be needed to investigate the apoptotic factors associated with the increased Bax/Bal-2 ratio as well as the activation of caspase-3, -9, and -8, and cleavage of poly-ADP ribose polymerase (PARP) in vitro. In our histological experiments and PCR analysis, the CWW 200 but not the CWW 50 group showed a significant difference from the BPH/vehicle group.

*C. wilfordii* has been shown to contain the acetophenones 2′,5′-dihydroxyacetophenone, 4′-hydroxyacetophenone, 2′,4′-dihydroxyacetophenone, 4′-hydroxy-3′-methoxyacetophenone, and cynandione A [52]. In our study, the HPLC chromatogram revealed that the CWW contained 4′-hydroxyacetophenone and 2′,4′-dihydroxyacetophenone, and the quantification showed their mean content was approximately 16 and 8 mg/g of extract, respectively. The active components of *C. wilfordii* have been reported to show anti-inflammatory, hepatoprotective, neuroprotective, and gastroprotective effects [21]. However, no studies have shown the beneficial effects of these active components on prostate hyperplasia.

Medical therapies used in the treatment of BPH include α-blockers and 5ARIs. α-Blockers are often used as first-line therapies [53]. They can produce rapid and considerable improvement of symptoms within 3–4 days of treatment initiation. However, they cannot reduce the size of the enlarged prostate [54]. 5ARIs such as finasteride and dutasteride reduce the size of the prostate gland, taking pressure off the urethra, and making it easier to urinate. The therapeutic effect of this drug appears after long-term use [55]. Therefore, combination therapy with α-blockers and 5ARIs is more effective in relieving symptoms and effectively preventing progression from benign prostatic hyperplasia than treatment with either drug alone. This combination is particularly used when the risk of progression of enlarged prostate is high (prostate volume > 30 mL, PSA level > 1.4 ng/mL) [55].

However, the usefulness of these drugs is limited because of their side effects including gynecomastia, erectile dysfunction, hypotension, and asthenia [56]. Therefore, herbal medicines have been considered as potential treatments for BPH. Herbal medicines generally have lower side effects and, as such, numerous patients with BPH are currently exploring the use of complementary and alternative medicines. Therefore, we attempted to identify an alternative drug for curing BPH using natural sources. To the best of our knowledge, this is the first study to report the effects of *C. wilfordii* in a BPH rat model.

## 5. Conclusions

In conclusion, oral administration of CWW at a dose of 200 mg/kg in a BPH rat model decreased the prostate weight and 5α-reductase and AR activities, as well as PSA, testosterone, and DHT levels in the serum and prostate. CWW at a dose of 200 mg/kg ameliorated the BPH-induced increase in Bcl-2 expression. However, the underlying molecular mechanisms remain to be elucidated. These findings indicate that 200 mg/kg CWW may effectively slow the progression of BPH and could be a potential herbal agent for BPH treatment, although more research studies including clinical trials are needed to confirm these findings. Furthermore, additional studies to ensure the safety of this substance are also required before it can be used in humans.

## Figures and Tables

**Table 1:** Effects of aqueous *Cynanchum wilfordii* extract (CWW) on body weight gain, prostate weight, and growth inhibition

Group	Body Weight Gain (g)	Prostate Weight (g)	Growth Inhibition (%)
Sham/vehicle	118.5 ± 5.0	0.82 ± 0.07	--
BPH/vehicle	41.5 ± 4.4	1.39 ± 0.07	--
BPH/finasteride	38.8 ± 4.2	1.06 ± 0.06 **	58.1 ± 10.7 **
BPH/CWW 50	33.6 ± 5.0	1.08 ± 0.07 *	54.5 ± 11.7 *
BPH/CWW 100	39.9 ± 6.6	1.10 ± 0.05 *	51.8 ± 9.7 *
BPH/CWW 200	41.9 ± 6.0	1.11 ± 0.08 *	50.1 ± 14.4 *

All data are mean ± standard error of the mean (S.E.M.). ** *p <* 0.01 and * *p <* 0.05 vs. BPH/vehicle group. Growth inhibition = 100 − ((treated group-sham group)/(BPH group-sham group) × 100); BPH: benign prostatic hyperplasia; Sham/vehicle: sham-operated and phosphate-buffered saline (PBS), orally (p.o.); BPH/vehicle: testosterone: subcutaneous (s.c.) and PBS, p.o.; BPH/finasteride: testosterone, s.c. and finasteride 5 mg/kg, p.o.; BPH/CWW 50: testosterone, s.c. and CWW 50 mg/kg, p.o.; BPH/CWW 100: testosterone, s.c. and CWW 100 mg/kg, p.o.; BPH/CWW 200: testosterone, s.c. and CWW 200 mg/kg, p.o.
